# Diet: Cause or Consequence of the Microbial Profile of Cholelithiasis Disease?

**DOI:** 10.3390/nu10091307

**Published:** 2018-09-14

**Authors:** Isabel Gutiérrez-Díaz, Natalia Molinero, Ana Cabrera, José Ignacio Rodríguez, Abelardo Margolles, Susana Delgado, Sonia González

**Affiliations:** 1 Department of Functional Biology, University of Oviedo, C/Julián Clavería s/n, 33006 Oviedo, Asturias, Spain; igutidiaz@gmail.com; 2 Group Diet, Microbiota and Health, Instituto de Investigación Sanitaria del Principado de Asturias (ISPA), Avda. Roma s/n, 33011 Oviedo, Asturias, Spain; 3 Department of Microbiology and Biochemistry of Dairy Products, Dairy Research Institute of Asturias–Spanish National Research Council (IPLA-CSIC), Paseo Río Linares s/n, 33300 Villaviciosa, Asturias, Spain; natalia.molinero@ipla.csic.es (N.M.); amargolles@ipla.csic.es (A.M.); 4 General Surgery Service, Colorectal Surgery Unit, Cabueñes University Hospital, Calle Los Prados 395, 33394 Gijón, Asturias, Spain; acabreraper@hotmail.com (A.C.); rodriguezgarciaji@gmail.com (J.I.R.)

**Keywords:** diet, polyphenols, fiber, cholelithiasis, biliary microbiota

## Abstract

Recent dietary habits and lifestyle could explain the shaping of the gut microbiota composition and, in consequence, the increasing prevalence of certain pathologies. However, little attention has been paid to the influence of diet on microbiotas, other than the gut microbiota. This is important in cholelithiasis, given that changes in the production of bile acids may affect gallbladder microbial communities. Our aim was to assess the association between regular dietary intake and gallbladder microbial composition. Fourteen adults with cholelithiasis and 14 controls, sex‒age-matched and without gastrointestinal pathology, were included. Diet was assessed through a food frequency questionnaire and quantification of gallbladder microbiota sequences by Illumina 16S rRNA gene-based analysis. The cholelithiasic patients showed greater intake of potatoes and lower consumption of vegetables, non-alcoholic drinks, and sauces, which resulted in a lower intake of energy, lipids, digestible polysaccharides, folate, calcium, magnesium, vitamin C, and some phenolic compounds. Regarding the altered bile microorganisms in cholelithiasic patients, dairy product intake was negatively associated with the proportions of *Bacteroidaceae* and *Bacteroides,* and several types of fiber, phenolics, and fatty acids were linked to the abundance of *Bacteroidaceae*, *Chitinophagaceae*, *Propionibacteraceae*, *Bacteroides*, and *Escherichia‒Shigella*. These results support a link between diet, biliary microbiota, and cholelithiasis.

## 1. Introduction

In the last few years, solid scientific evidence has emerged supporting the view that dietary patterns are intimately linked to the composition and activity of the millions of microbes that inhabit along the gastrointestinal tract [1,2,3,4]. This close relationship is the result of a co-evolutionary process over almost half a billion years, whereby the diets of our ancestors, rich in polysaccharides and antioxidants, forced evolution towards a microbiota dominated by saccharolytic bacteria capable of extracting additional energy from food, in addition to offering other benefits for the health of the host [2,5,6,7]. However, during recent times, industrialized countries have undergone a profound change in their dietary habits, resulting in the abandonment of a dietary pattern characterized by the abundance of cereals, tubers, vegetables, and fruits, in favour of a more “Westernized” pattern, characterized by the high consumption of refined foods, meats, and other products of animal origin [2,8]. This, together with other factors related to current lifestyle, could explain the change in the gut microbiota composition and the increasing prevalence of certain pathologies in the population. However, very little attention has been paid to the influence of diet on microbiotas other than the gut microbiota. 

In this regard, in the aetiology of cholelithiasis, one of the most common biliary disorders in adults from developed countries [9], diet has long been recognized as an important risk factor. Some mechanisms, such as the modification of gallbladder motility or the alteration of the composition of bile salts, have been proposed [10] in order to explain this association. However, how dietary components influence this pathology remains unclear. The impact of saturated fat intake on gut microbiota by means of a change in the pool of biliary acids has recently been demonstrated, so it seems possible that diet-driven changes in the production of bile acids affect gut microbes that, in turn, trigger disease [11,12]. Also, the intake of certain types of fiber that may be significant predictors in the pathogenesis of cholelithiasis has been described [13,14]. In this regard, the activity of the gut microbiota could also be linked to the development of cholelithiasis, by altering the concentration of biliary lipids in bile [13] and/or increasing the faecal excretion of bile salts [15,16]. However, and to the best of our knowledge, there are no previous studies examining the association between the regular dietary intake and the microbial composition in this organ. At this moment there is extremely scarce and fragmented information about the microbiota present in the gallbladder. In the last years a few reports analysed the microbiota of the biliary tract and the gallbladder using culture independent techniques. It was shown that *Enterobacteriaceae* dominate the biliary tract of acute cholecystitis and gallstone patients [17,18]. Also, a Chinese study with gallbladder gallstones patients showed that the biliary microbiota was constituted mainly the phyla Actinobacteria, Bacteroidetes, Firmicutes, and Proteobacteria, with *Bacteroides* being the most abundant genus [19]. It was also observed that these four phyla dominated the human gallbladder microbiota of patients with gallbladder gallstones [20]. Apart from this, the description of the microbiota of the human gallbladder has been hampered by difficulties in accessing bile samples and the lack of optimal culture conditions adapted to the biliary ecosystem. However, this information would be of great interest for the identification of the specific dietary components associated with the composition of the gallbladder microbial communities. 

## 2. Subjects and Methods

### 2.1. Participants

The study sample comprised 14 patients with cholelithiasis (three males, 11 females; mean age 51.50 ± 14.10 years old) and 14 controls, sex‒age-matched without declared gastrointestinal pathology (three males, 11 females; mean age 46.50 ± 11.47 years old). Subject recruitment was carried out at the General and Digestive Surgery Service of Cabueñes Hospital (Gijon, Spain). Information on clinical manifestations was obtained from clinical records and by personal interviews. Exclusion criteria, for both groups, were previous diagnosis of allergy, diabetes type II, metabolic syndrome, or autoimmune diseases, as well as having undergone medical treatment with antibiotics or glucocorticoids during the previous three months.

Potential volunteers were informed of the objectives of the study. When they agreed to participate, one personal appointment was made to collect dietary information and another to obtain the biological samples. All determinations were performed with fully informed written consent from all participants involved in the study.

Ethical approval for this study (reference code AGL2013-44761-P; grant title “The human bile microbiota: ecology, functionality and relationship to diet and biliary disorders”) was obtained from the Bioethics Committee of Consejo Superior de Investigaciones Científicas (CSIC) and from the Regional Ethics Committee for Clinical Research (Servicio de Salud del Principado de Asturias, n°112/13) in compliance with the Declaration of Helsinki. The study did not interfere with patients’ normal care.

### 2.2. Nutritional Assessment

Dietary intake was registered using an annual, semi-quantitative Food Frequency Questionnaire (FFQ), detailing 54 items. During a personalized interview with expert dietitians, participants were asked, item by item, whether they usually ate each food and, if so, how much they ate. Methodological issues concerning dietary assessment were detailed [21]. Food intake was analysed for energy, macronutrients, folate, cholesterol, calcium, vitamin C, magnesium, and total dietary fibre content by using the nutrient Food Composition Tables developed by the “Centro de Enseñanza Superior de Nutrición Humana y Dietética” (CESNID) [22]. The intake of fatty acids was converted using the National Nutrient Database for Standard Reference from the United States Department of Agriculture [23]. Also, information about dietary fibre components (soluble and insoluble) was completed using Marlett et al.’s food composition tables [24]. These authors used the enzymatic‒chemical method developed by Theander et al. [25], in which pectin content is determined using a calorimetric assay, cellulose and hemicellulose by high-performance liquid chromatography (HPLC), and Klason lignin is estimated as the insoluble material after a Seaman acid hydrolysis [26]. The phenolic compound content in foods was estimated using the Phenol Explorer database, which contains detailed information of over 400 foods regularly consumed in European countries [27].

### 2.3. Food Groups

Food intake was analysed in 17 food groups according to CESNID classification [22] as follows: cereals: pastry, bread, pasta, and flours and grains; dairy products: milk, yogurt, dairy dessert, fresh, mature, and processed cheeses; fats and oils: olive oil, other oils (sunflower and corn), and solid fats; sugar and sugary products: sweets, chocolate, honey, and jam; vegetables: bulbs, mushrooms, roots, inflorescence, and stem and leaf vegetables; legumes: lentils, chickpeas, beans and peas; meat and derived products: poultry, red meat, processed meat, and others; fish: low omega-3 and high omega-3 fish, and other derivatives; fruits: fresh, dried, and canned fruits; non-alcoholic drinks: coffee, tea, soft drinks, and juice; alcohol drinks: spirits, wine, beer, and cider; seafood: crustaceans and molluscs. Other categories include eggs, potatoes, sauces, snacks, and nuts and seeds. 

### 2.4. Anthropometric Measures

Height was measured using a stadiometer with an accuracy of ±1 mm (Año-Sayol, Barcelona, Spain). The subjects stood barefoot, in an upright position and with the head positioned in the Frankfort horizontal plane. Weight was obtained on a scale with an accuracy of ±100 g (Seca, Hamburg, Germany). Body mass index (BMI) was calculated using the formula: weight (Kg)/height (m)^2^. 

### 2.5. Biochemical Analysis

Fasting blood samples were drawn by venipuncture after a 12-h fast and collected in separate tubes for serum and plasma. Samples were kept on ice and centrifuged (1000× *g*, 15 min) within 2–4 h after collection. Plasma and serum aliquots were kept at −20 °C until analyses were performed. Serum glucose, total cholesterol, high-density lipoproteins (HDL), low-density lipoprotein (LDL), and triglycerides were determined by standard methods.

### 2.6. Bile Sample Collection and Microbiota Analysis Based on Sequencing of 16S rDNA Amplicons

Bile samples were obtained from 14 patients diagnosed with cholelithiasis, who underwent surgical removal at Cabueñes Gijon University Hospital (Asturias, Spain) to eliminate gallstones from the gallbladder. Total DNA extraction from bile samples was performed according to a previously described method [28]. Partial 16S rRNA gene sequences were amplified from the extracted DNA using the primer´s pair Probio Uni and /Probio_Rev, which target the variable region V3 of the bacterial 16S rRNA gene, as previously described [29]. Samples were submitted to 2 × 250 bp paired-end sequencing by an Illumina MiSeq System (Illumina, San Diego, CA, USA). All quality-approved, trimmed, and filtered sequences were processed using the QIIME^TM^ v1 open source bioinformatic pipeline (http://qiime.org/) (Flagstaff, AZ, USA) and sequences were classified to the lowest possible taxonomic rank using QIIME and the SILVA database as a reference [30]. Full experimental details will be reported elsewhere and provided in a subsequent full article (Molinero et al., data not shown). Raw sequence data were deposited in the Sequence Read Archive (SRA) of the NCBI (https://www.ncbi.nlm.nih.gov/sra) under the accession numbers SRR6872880 to SRR6872883 and SRR6872887 to SRR6872896. 

### 2.7. Statistical Analyses

Statistical analyses were performed using IBM-SPSS version 24.0 (SPSS-Inc., Chicago, IL, USA). Goodness of fit to normal distribution was analysed with the Kolmogorov‒Smirnov test. When the distribution of variables was skewed, the natural logarithm of each value was used in the statistical test. The Mann‒Whitney test was used to evaluate the differences in continuous variables between patients with biliary cholelithiasis and control subjects and chi-squared analysis for the categorical ones. For data on biliary microbiota, only those microbial taxa with relative abundancies ≥0.5 and detectable in at least one subject were considered. The Spearman correlation method was used to elucidate the relationship between food groups and dietary compounds with major biliary microbiota. Heatmaps were generated under R version 3.4.2 package heatmap.2. The dietary compounds previously correlated to biliary microbiota were selected and placed into a stepwise regression analysis to explore their independent effect. The statistical parameters employed were β (standardized regression coefficient) and *R*^2^ (coefficient of multiple determinations). The conventional probability value for significance (0.05) was used in the interpretation of results. 

## 3. Results 

### 3.1. General Characteristics of the Study Sample

A general description of the studied variables in the cholelithiasic patients and the controls is presented in Table 1.

While non-significant differences are shown for age, BMI, and sedentary lifestyle among the groups, lower levels of serum glucose and HDL, and higher concentrations of triglycerides were observed in these patients. Regarding dietary habits, even though 57.2% of cholelithiasic patients reported an excellent or good appetite, most of them (64.3%) declared that they excluded some foodstuffs from their regular diet, with legumes, dairy products, red meat, and vegetables being the most commonly excluded. In consequence, a lower intake of vegetables, sauces, and non-alcoholic drinks, and a higher consumption of potatoes, was found in patients with cholelithiasis (Table 2).

These differences resulted in a lower intake of energy, some macro-(digestible carbohydrates, total lipids, and polyunsaturated fatty acids (PUFA)) and micronutrients (folate, calcium, magnesium, and vitamin C) in the cholelithiasic group, together with a decreased intake of certain phenolic compounds: flavonoids (anthocyanins, flavanones, flavones, and flavanols), lignans, phenolic acids (hydroxybenzoic, hydroxycinnamic and hydroxyphenylacetic acids), and tyrosols (Table 3).

### 3.2. Diet and Gallbladder Microbiota in Cholelithiasic Patients

**Figure 1:**
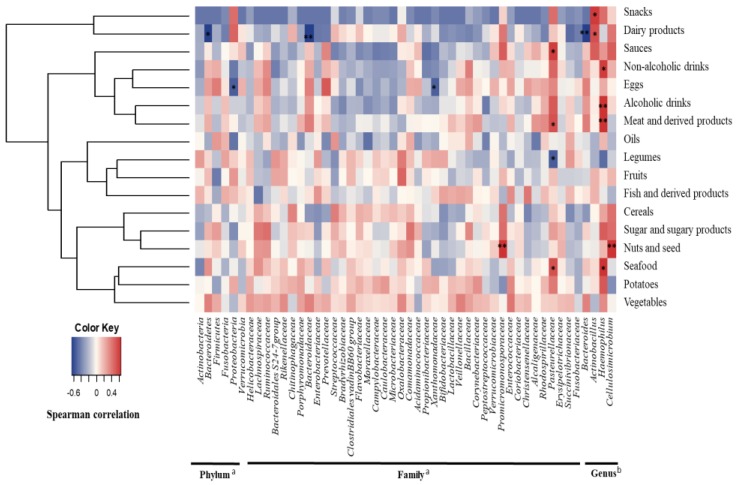
Spearman correlation between main food groups (g/day) from FFQ and gallbladder microbiota (%) in patients with cholelithiasis. ^a^ Phylum and families showing a relative abundance. ^b^ Genus belonging to families correlated with some food groups. Columns correspond to major phyla and families of biliary microbiota; rows correspond to the main food groups. Red and blue denote positive and negative association, respectively. The intensity of the colours represents the degree of association between the food groups and biliary microbiota as measured by the Spearman’s correlations, and dots indicate significant associations. * *p* < 0.05; ** *p* ≤ 0.01.

**Figure 2:**
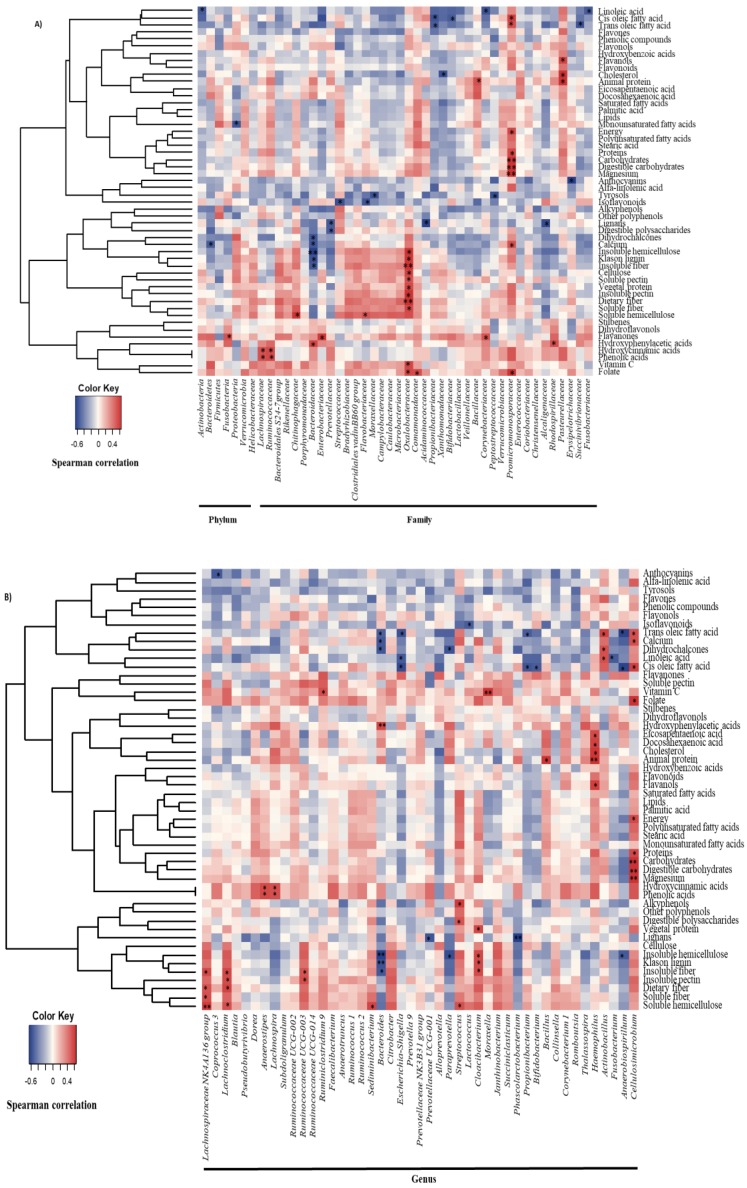
Spearman correlation between macro-, micronutrients, and bioactive compounds from the FFQ and gallbladder bacterial taxa in patients with cholelithiasis. (**A**) Phylum and families with a relative abundance. (**B**) Genera belonging to families correlated with some dietary compounds. Columns correspond to major phyla and families of biliary microbiota; rows correspond to the macro-, micronutrients and bioactive compounds. Red and blue denote positive and negative association, respectively. The intensity of the colours represents the degree of association between the dietary components and biliary microbiota as measured by the Spearman’s correlations, and dots indicate significant associations. * *p* < 0.05; ** *p* ≤ 0.01.

In order to explore the relationship between diet and the major members of the gallbladder microbiota, Spearman correlation analyses were conducted (Figure 1 and Figure 2). The intake of dairy products was negatively correlated to the abundance of the phylum *Bacteroidetes*, the family *Bacteroidaceae* and the genus *Bacteroides*; eggs were inversely associated with the proportions of *Proteobacteria,* and particularly with the family *Xanthomonadaceae*; and seafood and meats showed positive associations with the *Pasteurellaceae* family, and specifically with the genus *Haemophilus.* Moreover, *Pasteurellaceae* correlated directly with sauces, and the intake of legumes was negatively correlated with the relative abundance of this bacterial family (Figure 1). In addition, several dietary compounds were linked to the levels of different biliary microorganisms (Figure 2A,B).

At this point, given the high correlation between all the assessed variables, an additional stepwise regression analysis was conducted to explore the relative importance of dietary compound intake on the gallbladder microbiota (Table 4).

From the compounds previously associated with bile microbiota, linoleic acid was found to be an independent contributor to levels of *Actinobacteria,* and *Escherichia‒Shigella*, as well as the intake of *trans* oleic acids to *Propionibacteriaceae* and *Propionibacterium,* and another genus of the *Pasteurellaceae* family: *Actinobacillus*. Whereas *Bacteroidaceae* and *Bacteroides* variations were explained, in part, by the intake of insoluble fiber and hydroxyphenylacetic acids, the relative abundance of *Promicromonosporaceae* and *Cellulosimicrobium* was associated with magnesium and digestible carbohydrate intake. *Lachnoclostridium* levels (from the family *Lachnospiraceae*) were positively associated with dietary fiber intake, a family previously linked to butyrate production [31], while the proportions of *Chitinophagaceae,* and in particular *Sediminibacterium* genus [belonging to the *Fibrobacteres‒Chlorobi‒Bacteroidetes* superphylum (FCB group)], showed a direct relationship with soluble hemicellulose consumption. Also, insoluble fibers were found to be independent contributors to this group of bacteria, specifically the *Flavobacteriaceae* family and the genus *Cloacibacterium,* and insoluble pectin was also found to be an independent contributor to the *Oxalobacteraceae* family (belonging to the β-proteobacteria). Whereas the family *Lachnospiraceae* and the genus *Lachnospira* presented a direct association with hydroxycinnamic acids, the intake of vitamin C was related to the levels of the Gram-negative *Moraxella* and the Gram-positive *Ruminoclostridium* 9 (Table 4). 

## 4. Discussion

Although since the 70’s several surveys have been conducted in order to identify the existence of differences in the diets of subjects with cholelithiasis [32,33,34], the links between dietary factors and the development of the pathology are far from being completely understood. Compelling evidence has shed some light on the existence of microorganisms associated with the occurrence of this pathology. In this regard, it has been demonstrated that strains of *Salmonella* or *Listeria* monocytogenes are able to grow and survive within the gallbladder. In fact, the gallbladder can act as a reservoir for these bacteria, and one of the few bacteria frequently associated with gallstones is *Salmonella enterica* [35,36,37]. Furthermore, attempts have been made to analyse the biliary tract microbiota potentially associated with gallstones [19,38], using next-generation sequencing methods. Due to the crucial role described for gastrointestinal microbiota in human health, these findings generated novel interest regarding dietary factors able to modulate biliary associated microbial communities. 

To our knowledge, there is no consensus on defining a pro-lithiasic diet. While some previous studies have reported an increase in energy intake in cholelithiasic patients [39], others described the inverse situation [40]. As the relationship between energy intake and gallstone formation has been closely linked to the presence of obesity, this could be one of the differential factors between studies [41]. In our sample, while it has been observed a lower energy intake in the cholelithiasic group, no differences were found in BMI. Given that our patients had been diagnosed at least one year before recruitment, we propose the existence of a reduction in energy intake during this time, because of the exclusion of some foods that could be associated with the symptoms of this pathology [32], such as vegetables, non-alcoholic drinks, and sauces, and the reduction in the appetite reported by 42.8% of the sample, as has been shown in Table 1. Although the mean BMI of patients was indicative of moderate overweight, the findings presented herein do not support the role of obesity as a cholelithiasic risk factor, given the absence of differences with respect to the control group. Also, the lower level of HDL and the higher level of triglycerides observed (Table 1) were consistent with evidence supporting the potential role of cholesterol as a precursor in the formation of micelles with bile salts and phospholipids [41,42,43,44].

Analysing the global intake of the major food groups in the sample, a lower intake of vegetables has been found in the group with cholelithiasis [32] (Table 2). For decades, the protective effect of fruits and vegetables in the formation of gallstones was focused on their content of insoluble fibers, mainly lignin, which modifies the turnover of bile salts and cholesterol by means of reducing the lithogenic effect of the diet [45]. Our data do not support the existence of significant differences in the intake of fiber, neither in total nor by subclasses, between groups; nevertheless, a lower intake of most of the evaluated phenolic compounds has been observed in cholelithiasis (Table 3), suggesting a possible inverse association of these compounds in the pathology. Since foods are mixtures of bioactive compounds that could affect microbiota, there is no doubt about the complexity of analysing the associations for these components. For this reason, including the intake of fibers and polyphenols and their combined effect on biliary microbiota is probably the most novel aspect of this study. In addition, both fiber and polyphenol intake has been related in the patient group to the concentration of some of the microorganisms, whose proportion has been found to be altered in the bile of patients with lithiasis or primary sclerosing cholangitis (*Fusobacteria, Bacteroidaceae, Chitinophagaceae, Prevotellaceae*, and *Bacteroides*) [19,31,46], hence it is probable that microbiota could play a role in the protective effect proposed for vegetable sources. In this regard, from all the evaluated compounds hydroxyphenylacetic acid and insoluble fiber were pointed to as independent predictors of *Bacteroidaceae* genus *Bacteroides,* shown to be increased in this pathology [19], whereas cholesterol and *trans* oleic acid were found to be inversely related to the proportions of *Xanthomonadaceae* and *Propionibacteraceae*, respectively (Table 4). As the proportion of these latter microorganisms was found to be reduced in cholelithiasis as compared with healthy liver donors (Molinero et al., unpublished results), our results support the novel hypothesis proposed by Islam et al., suggesting that high-fat diets increase the secretion of bile acids showing strong antimicrobial activity [47]. In order to complete the overview of the role of diet in the gallbladder microbiota, it is worth highlighting the positive association found between the genera *Haemophillus* with the intake of seafood, meat, and drinks (Figure 1). This bacterial genus seems to be directly associated with the intake of flavanols, animal protein, cholesterol, and eicosapentaenoic and docosahexaenoic fatty acids (Figure 2). 

The current study is the first to establish a link between biliary microbiota, diet, and gallstone formation. At present, the information about these interactions is limited. Bacterial bile and cholesterol metabolism by the gut communities and its influence on health is already known [48], as well as the effect of diet on bile acid metabolism and gut microbiota composition [11,49]. However, the origin or even the existence of the bile microbiota is more controversial, with only a few studies focusing on that [18,38,46]. The present manuscript represents a proof-of-concept study of this previously unexplored scenario. Some of the findings herein presented, such as the association of certain phenolic compounds with *Bacteroides* and *Lachnospira*, or between vitamin C and *Moraxella* and *Ruminoclostridium,* may pave the way for future research projects focused on this gastrointestinal pathology. One of the limitations is the lower sample size, which could somewhat hamper our ability to detect significant associations by reducing the power of the statistical tests. Although a stepwise regression analysis has been carried out in order to avoid the high degree of correlation between the analysed variables, it is not possible to establish directionality in the relationship between the diet and the microbiota. The associations between these two variables have been carried out only in the group of cholelithiasic patients, given the impossibility of obtaining gallbladder samples from healthy volunteers. Even considering the above limitation, the knowledge generated in this work allows us to establish a new hypothesis that must be corroborated in the future. If these preliminary data are confirmed, they could be the first step in the rational design of diets adapted to this hepatobiliary disease. 

## Figures and Tables

**Table 1:** General characteristics of patients with cholelithiasis and control subjects.

Variables	Patients with Cholelithiasis (*n* = 14)	Control Subjects (*n* = 14)
Age (year)	51.50 ± 14.10	46.50 ± 11.47
Female (%)	78.6	78.6
BMI (kg/m^2^)	26.38 ± 6.04	26.00 ± 3.92
Drug use (%)	50.0	28.6
Probiotics consumption (%)	14.3	35.7
Vitamin and mineral supplements consumption (%)	0.0	35.7 **
Fiber supplementation (%)	0.0	14.3
Ethanol (g/day)	3.98 ± 4.98	5.64 ± 6.70
Current smoker (%)	14.3	14.3
Sedentary lifestyle (%)	50.0	50.0
Serum glucose (mg/dL)	79.14 ± 17.65	91.00 ± 9.83 *
Serum triglycerides (mg/dL)	129.14 ± 84.53	77.00 ± 33.63 *
HDL (mg/dL)	49.93 ± 14.79	61.21 ± 11.18 *
LDL (mg/dL)	117.07 ± 37.78	129.79 ± 35.22
Serum total cholesterol (mg/dL)	192.79 ± 55.33	206.71 ± 39.47

Results derived from Mann‒Whitney U test were presented as estimated marginal mean ± standard deviation Differences in categorical variables were examined using chi-squared analysis and presented as percentage (%). BMI: body mass index. HDL: high-density lipoprotein. LDL: low-density lipoprotein. * *p* ≤ 0.05, ** *p* ≤ 0.01.

**Table 2:** Dietary habits and daily intake of the major food groups in patients with cholelithiasis and control subjects.

Dietary Habits	Patients with Cholelithiasis (*n* = 14)	Control Subjects (*n* = 14)
Appetite ^a^ (%)		
Excellent‒Good	57.2	-
Normal	28.5	-
Regular	14.3	-
Chewing problems ^a^ (%)	0.0	-
Special diet ^a^ (%)	35.7	-
Omit foodstuffs from diet ^a^ (%)	64.3	-
Legumes	35.7	-
Dairy products	14.3	-
Red meat and meat products	64.3	-
Vegetables	14.3	-
No. of subjects reporting a change in food habits ^a^ (%)	28.6	-
**Food Groups (g/day)**		
Cereals	121.14 ± 74.97	142.45 ± 68.39
Dairy products	415.86 ± 206.42	522.15 ± 227.64
Eggs	13.78 ± 13.38	18.60 ± 11.13
Fats and oils	23.30 ± 12.44	31.16 ± 16.99
Sugar and sugary products	16.74 ± 20.78	17.71 ± 17.45
Vegetables	200.17 ± 85.11	318.16 ± 147.14 *
Potatoes	56.46 ± 30.51	33.27 ± 23.51 *
Legumes	27.10 ± 20.82	32.60 ± 26.65
Fruits	244.90 ± 169.37	320.66 ± 151.02
Meat and derived products	129.44 ± 63.15	109.76 ± 34.00
Fish and derived products	60.49 ± 26.76	65.43 ± 31.55
Seafood	12.53 ± 14.30	20.90 ± 24.76
Non-alcoholic drinks	96.94 ± 142.15	369.62 ± 235.93 **
Alcoholic drinks	86.22 ± 110.36	134.96 ± 167.97
Nuts and seed	20.52 ± 55.29	10.72 ± 14.83
Sauces	2.82 ± 4.03	8.29 ± 7.82 *
Snack	2.86 ± 10.69	2.60 ± 4.39

Results derived from Mann‒Whitney U test are presented as estimated marginal mean ± SD and percentage (%). “-” (not measured). ^a^ Variables obtained only for patients with cholelithiasis. * *p* ≤ 0.05, ** *p* ≤ 0.01.

**Table 3:** Macro-, micronutrients, and bioactive compounds in patients with cholelithiasis and control subjects.

	Unadjusted		Adjusted	
Dietary Components	Patients with Cholelithiasis (*n* = 14)	Control Subjects (*n* = 14)	Patients with Cholelithiasis (*n* = 14)	Control Subjects (*n* = 14)
Energy (kcal/day)	1660.25 ± 659.28	2079.06 ± 515.84 *	-	-
Carbohydrates (g/day)	170.16 ± 72.05	209.71 ± 51.60	190.12 ± 72.05	189.74 ± 51.60
Digestible carbohydrates	82.04 ± 41.35	114.03 ± 30.63 *	91.77 ± 41.35	104.30 ± 30.63
Digestible polysaccharides	80.46 ± 38.54	84.29 ± 37.14	91.39 ± 38.54	73.36 ± 37.14
Proteins (g/day)	87.20 ± 21.78	100.88 ± 19.97	93.52 ± 21.78	94.56 ± 21.78
Animal	58.22 ± 16.68	66.05 ± 17.71	60.61 ± 16.68	63.63 ± 17.71
Vegetable	25.02 ± 16.46	31.78 ± 18.09	29.44 ± 16.46	27.37 ± 18.09
Lipids (g/day)	66.65 ± 36.77	88.06 ± 34.30 *	78.31 ± 36.77	76.39 ± 34.30
SFA	18.23 ± 9.18	25.78 ± 10.05	21.04 ± 9.18	22.98 ± 10.05
Palmitic fatty acid	8.60 ± 4.05	10.73 ± 4.37	9.82 ± 4.05	9.51 ± 4.37
Stearic fatty acid	3.46 ± 2.57	3.82 ± 1.55	4.11 ± 2.57	3.17 ± 1.55 *
MUFA	29.38 ± 13.27	39.10 ± 16.59	33.56 ± 13.27	34.92 ± 16.59
Cis oleic fatty acid	1.28 ± 1.01	1.55 ± 1.07	1.43 ± 1.01	1.41 ± 1.07
Trans oleic fatty acid	0.12 ± 0.14	0.14 ± 0.14	0.13 ± 0.14	0.13 ± 0.14
PUFA	13.52 ± 18.90	15.19 ± 10.25 *	17.54 ± 18.90	11.18 ± 10.25
Linoleic fatty acid	0.19 ± 0.11	0.63 ± 0.95	0.20 ± 0.11	0.63 ± 0.95
Alfa-linolenic fatty acid	0.09 ± 0.08	0.11 ± 0.07	0.10 ± 0.08	0.10 ± 0.07
Docosahexaenoic fatty acid	0.21 ± 0.13	0.23 ± 0.14	0.20 ± 0.13	0.23 ± 0.14
Eicosapentaenoic fatty acid	0.09 ± 0.05	0.11 ± 0.08	0.09 ± 0.05	0.11 ± 0.08
Folate (µg/day)	301.26 ± 148.29	446.34 ± 111.00 **	330.25 ± 148.29	417.36 ± 111.00 *
Cholesterol (mg/day)	243.47 ± 126.33	303.03 ± 86.00	255.53 ± 126.33	290.97 ± 86.00
Calcium (mg/day)	840.95 ± 278.81	1260.04 ± 307.79 **	916.85 ± 278.81	1184.14 ± 307.79 **
Magnesium (mg/day)	348.44 ± 270.31	403.52 ± 110.17 *	403.30 ± 270.31	348.66 ± 110.17
Vitamin C (mg/day)	111.50 ± 61.73	193.63 ± 66.52 **	109.75 ± 61.73	195.39 ± 66.52 **
Phenolic compounds (mg/day)	946.11 ± 473.35	1780.48 ± 899.58 **	1025.80 ± 473.35	1700.78 ± 899.58 *
Flavonoids	211.79 ± 141.50	499.54 ± 393.48 *	248.44 ± 141.50	462.89 ± 393.48
Anthocyanins	7.45 ± 10.69	26.07 ± 35.29 *	5.49 ± 10.69	28.03 ± 35.29 *
Dihydroflavonols	1.04 ± 1.78	1.33 ± 2.84	1.00 ± 1.78	1.38 ± 2.84
Dihydrochalcones	1.78 ± 1.98	2.62 ± 3.97	1.94 ± 1.98	2.46 ± 3.97
Flavanols	167.01 ± 122.61	360.71 ± 397.60	206.56 ± 122.61	321.16 ± 397.60
Flavanones	16.87 ± 33.49	43.54 ± 31.14 **	13.55 ± 33.49	46.86 ± 31.14 *
Flavones	0.54 ± 0.57	5.15 ± 4.15 **	0.20 ± 0.57	5.49 ± 4.15 **
Flavonols	17.06 ± 10.18	37.27 ± 18.16 **	16.71 ± 10.18	37.63 ± 18.16 *
Isoflavonoids	0.03 ± 0.03	22.84 ± 76.64	3.00 ± 0.03	19.88 ± 76.64
Lignans	0.40 ± 0.61	1.14 ± 0.60 **	0.49 ± 0.61	1.05 ± 0.60 *
Phenolic acids	122.64 ± 153.59	336.09 ± 301.89 **	145.93 ± 153.59	312.80 ± 301.89
Hydroxybenzoic acids	7.20 ± 9.24	40.61 ± 28.38 **	7.56 ± 9.24	40.25 ± 28.38 **
Hydroxycinnamic acids	115.37 ± 152.11	294.28 ± 292.73 **	138.21 ± 152.11	271.44 ± 292.73
Hydroxyphenylacetic acids	0.07 ± 0.09	0.98 ± 1.61 **	0.14 ± 0.09	0.91 ± 1.61
Stilbenes	0.68 ± 1.13	0.99 ± 1.85	0.64 ± 1.13	1.04 ± 1.85
Other polyphenols	9.61 ± 12.99	42.22 ± 32.60 **	10.65 ± 12.99	41.18 ± 32.60 **
Alkyphenols	4.75 ± 8.53	18.92 ± 29.75	4.56 ± 8.53	19.11 ± 29.75
Tyrosols	4.05 ± 6.41	19.34 ± 10.83 **	5.29 ± 6.41	18.11 ± 10.83 **
Dietary fiber (g/day)	20.20 ± 8.47	24.21 ± 9.87	22.05 ± 8.47	22.36 ± 9.87
Soluble	2.28 ± 1.18	2.56 ± 0.94	2.42 ± 1.18	2.41 ± 0.94
Pectin	0.74 ± 0.49	0.90 ± 0.43	0.74 ± 0.49	0.90 ± 0.43
Hemicellulose	1.43 ± 0.87	1.51 ± 0.66	1.57 ± 0.87	1.36 ± 0.66
Insoluble	12.77 ± 6.25	15.60 ± 7.40	13.44 ± 6.25	14.92 ± 7.40
Pectin	1.65 ± 0.84	1.84 ± 0.77	1.71 ± 0.84	1.77 ± 0.77
Hemicellulose	4.61 ± 3.04	5.13 ± 3.01	4.99 ± 3.04	4.75 ± 3.01
Klason lignin	1.68 ± 1.13	2.18 ± 0.85	1.84 ± 1.13	2.02 ± 0.85
Cellulose	5.10 ± 2.03	6.26 ± 3.08	5.31 ± 2.03	6.03 ± 3.08

Results derived from Mann‒Whitney U test (unadjusted) or multivariate analysis adjusted by energy (adjusted). Variables are presented as estimated marginal mean ± SD. SFA, saturated fatty acids. MUFA, monounsaturated fatty acids. PUFA, polyunsaturated fatty acids. “-” (not measured). * *p* ≤ 0.05, ** *p* ≤0.01.

**Table 4:** Results obtained from a stepwise regression analysis in order to identify the dietary compounds predictors of the relative abundance of the major gallbladder bacterial communities (%).

	Predictors	*R* ^2^	β	*p* Value
**Phylum**				
*Actinobacteria*	Linoleic fatty acid (g/day)	0.360	−0.600	0.023
**Family**				
*Bacteroidaceae*	Hydroxyphenylacetic acids (mg/day)	0.709	0.722	0.001
	Insoluble fiber (g/day)		−0.401	0.031
*Chitinophagaceae*	Soluble hemicellulose (g/day)	0.524	0.724	0.003
*Flavobacteriaceae*	Insoluble hemicellulose (g/day)	0.393	0.627	0.016
*Lachnospiraceae*	Hydroxycinnamic acids (mg/day)	0.383	0.619	0.018
*Oxalobacteriaceae*	Insoluble pectin (g/day)	0.728	0.853	0.000
*Promicromonosporaceae*	Magnesium (mg/day)	0.962	1.116	0.000
	Digestible carbohydrates (g/day)		−0.313	0.002
*Propionibacteriaceae*	Trans oleic fatty acid (g/day)	0.378	−0.615	0.019
**Genus**				
*Actinobacillus*	Trans oleic fatty acid (g/day)	0.321	0.566	0.035
*Bacteroides*	Hydroxyphenylacetic acids (mg/day)	0.709	0.722	0.001
	Insoluble fiber (g/day)		−0.401	0.031
*Cellulosimicrobium*	Magnesium (mg/day)	0.962	1.161	0.000
	Digestible carbohydrates (g/day)		−0.313	0.002
*Cloacibacterium*	Insoluble fiber (g/day)	0.549	0.741	0.002
*Escherichia-Shigella*	Linoleic acid (g/day)	0.286	−0.535	0.049
*Lachnosclostridium*	Dietary fiber (g/day)	0.316	0.562	0.036
*Lachnospira*	Hydroxycinnamic acids (mg/day)	0.894	0.946	0.000
*Moraxella*	Vitamin C (mg/day)	0.652	0.807	0.000
*Propionibacterium*	Trans oleic fatty acid (g/day)	0.379	−0.616	0.019
*Ruminoclostridium 9*	Vitamin C (mg/day)	0.430	0.656	0.011
*Sediminibacterium*	Soluble hemicellulose (g/day)	0.533	0.730	0.003

Results derived from stepwise regression analysis; *R*^2^: coefficient of multiple determinations; β: standardized regression coefficient. Only significant results are presented.

## References

[1] Wu G.D., Chen J., Hoffmann C., Bittinger K., Chen Y.Y., Keilbaugh S.A., Bewtra M., Knights D., Walters W.A., Knight R. (2011). Linking long-term dietary patterns with gut microbial enterotypes. Science.

[2] De Filippo C., Cavalieri D., Di Paola M., Ramazzotti M., Poullet J.B., Massart S., Collini S., Pieraccini G., Lionetti P. (2010). Impact of diet in shaping gut microbiota revealed by a comparative study in children from Europe and rural Africa. Proc. Natl. Acad. Sci. USA.

[3] Gutiérrez-Díaz I., Fernández-Navarro T., Sánchez B., Margolles A., González S. (2016). Mediterranean diet and faecal microbiota: A transversal study. Food Funct..

[4] De Filippis F., Pellegrini N., Vannini L., Jeffery I.B., La Storia A., Laghi L., Serrazanetti D.I., Di Cagno R., Ferrocino I., Lazzi C. (2016). High-level adherence to a Mediterranean diet beneficially impacts the gut microbiota and associated metabolome. Gut.

[5] Martens E.C., Lowe E.C., Chiang H., Pudlo N.A., Wu M., McNutly N.P., Abbott D.W., Henrissat B., Gilbert H.J., Bolam D.N. (2011). Recognition and degradation of plant cell wall polysaccharides by two human gut symbionts. PLoS Biol..

[6] Martens E.C., Koropatkin N.M., Smith T.J., Gordon J.I. (2009). Complex glycan catabolism by the human gut microbiota: The bacteroidetes sus-like paradigm. J. Biol. Chem..

[7] Wright D.P., Rosendale D.I., Roberton A.M. (2000). Prevotella enzymes involved in mucin oligosaccharide degradation and evidence for a small operon of genes expressed during growth on mucin. FEMS Microbiol Lett..

[8] Broussard J.L., Devkota S. (2016). The changing microbial landscape of Western society: Diet, dwellings and discordance. Mol. Metab..

[9] Portincasa P., Moschetta A., Palasciano G. (2006). Cholesterol gallstone disease. Lancet.

[10] Attili A.F., Scafato E., Marchioli R., Marfisis R.M., Festi D. (1998). Diet and gallstones in Italy: The cross-sectional MICOL results. Hepatology.

[11] Devkota S., Wang Y., Musch M.W., Leone V., Fehlner-Peach H., Nadimpalli A., Antonopoulos D.A., Jabri B., Chang E.B. (2012). Dietary-fat-induced taurocholic acid promotes pathobiont expansion and colitis in Il10^−^/^−^mice. Nature.

[12] Yokota A., Fukiya S., Islam K.B., Ooka T., Ogura Y., Hayashi T., Hagio M., Ishizuka S. (2012). Is bile acid a determinant of the gut microbiota on a high-fat diet?. Gut Microbes.

[13] Burkitt D.P. (1976). Economic development-not all bonus. Nutr. Today.

[14] Russell M., Hollingsworth D.F. (1973). Proceedings of the British Nutrition Foundation Research Conference on ‘Nutritional Problems in a Changing World-Nutrition in Britain Today and Tomorrow’. Nutritional Problems in a Changing World.

[15] Antonis A., Bersohn I. (1962). The influence of diet on fecal lipids in South African white and Bantu prisoners. Am. J. Clin. Nutr..

[16] Jenkins D.J., Hill M.S., Cummings J.H. (1975). Effect of wheat fiber on blood lipids, fecal steroid excretion and serum iron. Am. J. Clin. Nutr..

[17] Liu J., Yan Q., Luo F., Shang D., Wu D., Zhang H., Shang X., Kang X., Abdo M., Liu B. (2015). Acute cholecystitis associated with infection of *Enterobacteriaceae* from gut microbiota. Clin. Microbiol. Infect..

[18] Ye F., Shen H., Li Z., Meng F., Li L., Yang J., Chen Y., Bo X., Zhang X., Ni M. (2016). Influence of the biliary system on biliary bacteria revealed by bacterial communities of the human biliary and upper digestive tracts. PLoS ONE.

[19] Wu T., Zhang Z., Liu B., Hou D., Liang Y., Zhang J., Shi P. (2013). Gut microbiota dysbiosis and bacterial community assembly associated with cholesterol gallstones in large-scale study. BMC Genom..

[20] Saltykova I.V., Petrov V.A., Logacheva M.D., Ivanova P.G., Merzlikin N.V., Sazonov A.E., Ogorodova L.M., Brindley P.J. (2016). Biliary microbiota, gallstone disease and infection with *Opisthorchis felineus*. PLoS Negl. Trop. Dis..

[21] Cuervo A., Valdes L., Salazar N., De los Reyes-Gavilan C.G., Ruas-Madiedo P., Gueimonde M., Gonzalez S. (2014). Pilot study of diet and microbiota: Interactive associations of fibers and polyphenols with human intestinal bacteria. J. Agric. Food Chem..

[22] Centro de Enseñanza Superior de Nutricion Humana y Dietetica (CESNID) (2008). Tablas de Composición de Alimentos por Medidas Caseras de Consumo Habitual en España.

[23] United States Department of Agriculture (USDA) Agriculture Research Service, 2016 USDA National Nutrient Database for Standard References. http://www.ars.usda.gov/services/docs.htm?docid=8964.

[24] Marlett J.A., Cheung T.F. (1997). Database and quick methods of assessing typical dietary fiber intakes using data for 228 commonly consumed foods. J. Am. Diet. Assoc..

[25] Theander O., Westerlund E.A. (1986). Studies on dietary fiber. 3. Improved procedures for analysis of dietary fiber. J. Agric. Food Chem..

[26] Seaman J.F. (1945). Kinetics of wood saccharification: Hydrolysis of cellulose and decomposition of sugars in dilute acid at high temperature. Ind. Eng. Chem..

[27] Neveu V., Perez-Jimenez J., Vos F., Crespy V., Du C.L., Mennen L., Knox C., Eisner R., Cruz J., Wishart D. (2010). Phenol-Explorer: An online comprehensive database on polyphenol contents in foods. Database.

[28] Jimenez E., Sánchez B., Farina A., Margolles A., Rodríguez J.M. (2014). Characterization of the bile and gall bladder microbiota of healthy pigs. Microbiologyopen.

[29] Milani C., Hevia A., Foroni E., Duranti S., Turroni F., Lugli G.A., Sanchez B., Martin R., Gueimonde M., Van Sinderen D. (2013). Assessing the fecal microbiota: An optimized ion torrent 16S rRNA gene-based analysis protocol. PLoS ONE.

[30] Quast C., Pruesse E., Yilmaz P., Gerken J., Schweer T., Yarza P., Peplies J., Glöckner F.O. (2013). The SILVA ribosomal RNA gene database project: Improved data processing and web-based tools. Nucleic Acids Res..

[31] Meehan C.J., Beiko R.G. (2014). A phylogenomic view of ecological specialization in the *Lachnospiraceae*, a family of digestive tract-associated bacteria. Genome Biol. Evol..

[32] Sarles H., Chabert C., Pommeau Y., Save E., Mouret H., Gérolami A. (1969). Diet and cholesterol gallstones. A study of 101 patients with cholelithiasis compared to 101 matched controls. Am. J. Dig. Dis..

[33] Reid J.M., Fullmer S.D., Pettigrew K.D., Burch T.A., Bennett P.H., Miller M., Whedon G.D. (1971). Nutrient intake of Pima Indian women: Relationships to diabetes mellitus and gallbladder disease. Am. J. Clin. Nutr..

[34] Wheeler M., Hills L.L., Laby B. (1970). Cholelithiasis: A clinical and dietary survey. Gut.

[35] Hardy J., Francis K.P., DeBoer M., Chu P., Gibbs K., Contag C.H. (2004). Extracellular replication of *Listeria monocytogenes* in the murine gall bladder. Science.

[36] Hardy J., Margolis J.J., Contag C.H. (2006). Induced biliary excretion of *Listeria monocytogenes*. Infect. Immun..

[37] Gonzalez-Escobedo G., Marshall J.M., Gunn J.S. (2011). Chronic and acute infection of the gall bladder by *Salmonella Typhi:* Understanding the carrier state. Nat. Rev. Microbiol..

[38] Shen H., Ye F., Xie L., Yang J., Li Z., Xu P., Meng F., Li L., Chen Y., Bo X. (2015). Metagenomic sequencing of bile from gallstone patients to identify different microbial community patterns and novel biliary bacteria. Sci. Rep..

[39] Wheeler H.O. (1973). Pathogenesis of gallstones. Surg. Clin. N. Am..

[40] Sarles H., Hauton J., Lafont H., Teissier N., Planche N.E., Gerolami A. (1968). Effect of diet on the biliary cholesterol concentration in normals and gallstone patients. Clin. Chim. Act..

[41] Admirand W.H., Small D.M. (1968). The physicochemical basis of cholesterol gallstone formation in man. J. Clin. Investig..

[42] Small D.M., Rapo S. (1970). Source of abnormal bile in patients with cholesterol gallstones. N. Engl. J. Med..

[43] Paigen B., Carey M.C., King R.A., Rotter J.I., Motulsky A.G. (2002). Gallstone. The Genetic Basis of Common Diseases.

[44] Tsai C.J., Leitzmann M.F., Willett W.C., Giovannucci E.L. (2004). Long-term intake of dietary fiber and decreased risk of cholecystectomy in women. Am. J. Gastroenterol..

[45] Bloomfield D.K. (1963). Dynamics of cholesterol metabolism. I. Factors regulating total sterol biosynthesis and accumulation in the rat. Proc. Natl. Acad. Sci. USA.

[46] Pereira P., Aho V., Arola J., Boyd S., Jokelainen K., Paulin L., Auvinen P., Färkkilä M. (2017). Bile microbiota in primary sclerosing cholangitis: Impact on disease progression and development of biliary dysplasia. PLoS ONE.

[47] Islam K.B.M.S., Fukiya S., Hagio M., Fujii N., Ishizuka S., Ooka T., Ogura Y., Hayashi T., Yokota A. (2011). Bile acid is a host factor that regulates the composition of the cecal microbiota in rats. Gastroenterology.

[48] Gérard P. (2013). Metabolism of cholesterol and bile acids by the gut microbiota. Pathogens.

[49] Devkota S., Chang E.B. (2015). Interactions between diet, bile acid metabolism, gut microbiota, and Inflammatory Bowel Diseases. Dig. Dis..

